# Social Demography of Transitional Dietary Patterns in Thailand: Prospective Evidence from the Thai Cohort Study

**DOI:** 10.3390/nu9111173

**Published:** 2017-10-27

**Authors:** Keren Papier, Susan Jordan, Catherine D’Este, Cathy Banwell, Vasoontara Yiengprugsawan, Sam-ang Seubsman, Adrian Sleigh

**Affiliations:** 1 National Centre for Epidemiology and Population Health (NCEPH) and Department of Global Health, Research School of Population Health, ANU College of Health & Medicine, The Australian National University, Canberra 2601, Australia; Catherine.Deste@anu.edu.au (C.D.); Cathy.Banwell@anu.edu.au (C.B.); vasoontara.yieng@anu.edu.au (V.Y.); Adrian.Sleigh@anu.edu.au (A.S.); 2 Population Health Department, QIMR Berghofer Medical Research Institute, Brisbane 4006, Australia; Susan.Jordan@qimrberghofer.edu.au; 3 The School of Public Health, The University of Queensland, Brisbane 4006, Australia; 4 School of Medicine and Public Health, Faculty of Health and Medicine, The University of Newcastle, Newcastle 2308, Australia; 5 Centre for Research on Ageing, Health and Wellbeing, Research School of Population Health, The Australian National University, Canberra 2601, Australia; 6 Thai Health-Risk Transition Study, School of Human Ecology, Sukhothai Thammathirat Open University, Nonthaburi 11120, Thailand; sam-ang.seu@stou.ac.th

**Keywords:** socioeconomic status, diet patterns, Asian cohort, urban, nutrition transition, principle component analysis

## Abstract

In recent decades, a health-risk transition with changes in diet and lifestyle in low and middle-income countries (LMICs) led to an emergence of chronic diseases. These trends in Southeast Asian LMICs are not well studied. Here, we report on transitional dietary patterns and their socio-demographic predictors in Thai adults. Dietary data in 2015 were from a random sub-sample (*N* = 1075) of 42,785 Thai Cohort Study (TCS) members who completed all three TCS surveys (2005, 2009, 2013). Principle Component Analysis identified dietary patterns and multivariable linear regression assessed associations (Beta estimates (ß) and confidence intervals (CIs)) between socio-demographic factors and dietary intake pattern scores. Four dietary patterns emerged: Healthy Transitional, Fatty Western, Highly Processed, and Traditional. In women, higher income (≥30,001 Baht/month vs. ≤10,000) and managerial work (vs. office assistant) was associated with lower scores for Traditional (ß = −0.67, 95% CI −1.15, −0.19) and Fatty Western diets (ß = −0.60, 95% CI −1.14, −0.05), respectively. University education associated with lower Highly Processed (ß = −0.57, 95% CI −0.98, −0.17) and higher Traditional diet scores (ß = 0.42, 95% CI 0.03, 0.81). In men and women, urban residence associated with higher Fatty Western and lower Traditional diets. Local policy makers should promote healthy diets, particularly in urban residents, in men, and in low-SEP adults.

## 1. Introduction

Rapid economic growth in low and middle-income countries (LMICs) has resulted in transformation of food systems and diets with a remarkable increase in the intake of animal fats and sugars. Concurrent urbanization and decreased physical activity is leading to increased body size and an epidemic of unfamiliar non-communicable diseases (NCDs), including diabetes, hypertension, and ischemic heart disease [1].

Collectively, these shifts in environment, behaviour, and disease, together with the health system response, have been termed the “health-risk transition” [2]. The nutritional components of the health-risk transition have been long recognized as a “nutrition transition” [3]. The changes in lifestyle behaviours and disease outcomes generally occur first in urban residents who have a high socio-economic position (SEP) [4]. Indeed, some studies from developing LMICs have found that urban, high SEP individuals have a higher prevalence of NCDs and are more likely to consume diets that are associated with an increased NCD risk than rural, low-SEP individuals [5,6]. However, recent evidence suggests that as more economic development occurs, the transition deepens and unhealthy diets and the NCDs shift to rural and low SEP individuals [6,7,8,9,10,11] This pattern resembles what is commonly observed in high income countries (HICs) that are at the advanced stage of the health-risk transition [12,13].

Thailand is a LMIC that has achieved substantial economic growth in recent decades [14] and now has an emerging type 2 diabetes (T2DM) epidemic [15]. The socio-demographic determinants of dietary intake in Southeast Asian countries like Thailand are not well understood. A few small, cross-sectional studies suggest that social differences in diet and health outcomes among Thai adults are beginning to resemble what is commonly observed in countries at the later stages of the nutrition transition (e.g., higher prevalence of obesity in rural and low SEP women; higher consumption of healthy foods in wealthy men and women) [16,17]. However, it is unclear how socio-demographic factors associate with dietary patterns in Thai adults. Understanding the drivers of dietary patterns will allow for the development of more targeted public health interventions that are aimed at controlling the T2DM epidemic. In this study, we identify major dietary patterns and examine the associations between socio-demographic factors and dietary patterns in a cohort of Thai adults.

## 2. Materials and Methods

Members of the Thai Cohort Study (TCS) were the source population for this research. The cohort is a prospective study of the “health-risk transition” among Sukhothai Thammathirat Open University (STOU) students that are residing nationwide [18]. In 2005, all 200,000 enrolled students were invited to participate and were mailed a questionnaire covering a wide array of variables including socio-demographic, health and lifestyle factors, and health outcomes. These were distance learning students, mostly part time and a little more urbanized than the national population, using education for self-improvement. As such, they are likely to experience the “health-risk transition” ahead of their fellow Thais. A total of 87,151 (44%) returned the completed questionnaire and formed the baseline cohort. Four years later (2009), 60,569 (69%) were successfully followed up, and of these, 42,785 (71%) were followed again in 2013.

### 2.1. Participant Selection

TCS members who completed all three questionnaires (2005, 2009, and 2013) were eligible for the current study (N = 42,785). Previous experience with this cohort suggests that around half of all TCS members invited to participate in sub studies respond [19]. In order to achieve our desirable sample size of ~1000 (see Statistical methods), we invited a random sample of 2400 TCS members to complete an additional mail-out dietary survey in 2015 expecting that approximately ~1100 participants would be successfully followed up. 

### 2.2. Dietary Intake

Dietary intake was assessed in 2015 using the validated Thai National Health Examination Survey food frequency questionnaire (FFQ) [20]. The participants were asked to indicate the frequency of consumption of each food item on average with one of seven response categories ranging from “don’t eat at all” to “more than once per day”. FFQ responses for each item were converted into daily intake equivalents as follows: “don’t eat at all” = 0, “less than once per month” (0.5/30 = 0.02), “1–3 times per month” = 0.07, “1–3 times per week” = 0.28, “4–6 times per week” = 0.71, “once per day” = 1, or “more than once per day” = 2.5. Participants were excluded from this study if responses to >10% of food consumption items were missing while all of the other missing FFQ items were considered as not consumed [21]. All of the 44 food items were allocated into 30 separate food groups according to nutritional content, culinary use, and previous dietary pattern studies [17] (Table S1).

### 2.3. Socio-Economic Position

We used three measures of socio-economic position (SEP): monthly income, occupation, and highest level of attained education. Information on these measures was collected in the 2013 questionnaire. Occupation was included because personal monthly income is affected by earning disparities between Thai men and women [22]. Using occupation as a measure of SEP may help to detect differences in low and high SEP women that personal monthly income might not be able to discern due to the low number of women in the high income bracket. Data for all three SEP measures were collected in the 2013 follow-up questionnaire. Personal monthly income (Baht) was reported in categories and classified as: <10,000 (<295 USD), 10,001–20,000 (295–590 USD), 20,001–30,000 (>590–880 USD), or ≥30,001 (>880 USD). Level of attained education was categorized as having or not having a university degree. Data on occupation were reported in categories and further classified as: manual worker (e.g., labourers), office assistant, skilled worker (e.g., carpenter, hairdresser), professional (e.g., doctor, accountant), and manager (middle or senior). 

### 2.4. Demographic Factors

Information on the location of current residence was collected in the 2005, and again in the 2013 follow-up questionnaires. In both questionnaires, residence was recorded as rural or urban. We combined the data for 2005 and 2013 and converted this measure into four categories based on residence reported in 2005 and in 2013: rural residence in both 2005 and 2013; rural residence in 2005, urban residence in 2013; urban residence in both 2005 and 2013; and, urban residence in 2005, rural residence in 2013. 

### 2.5. Statistical Methods

Since dietary intake in this cohort varies substantially by sex, especially for transitional foods, we performed all of the analyses separately for men and women [23].

#### 2.5.1. Dietary Patterns

Dietary patterns were identified using principle component analysis (PCA). We determined the number of patterns to retain based on their eigenvalues (>1.0) (pointing to factors explaining more of the total variance than each original variable), using scree plots, and according to the interpretability of the identified pattern. The retained patterns were then orthogonally rotated to obtain a simpler factor structure and enhance their interpretability [24]. Food items with an absolute factor loading >0.30 or <−0.30 were considered as substantial contributors. Patterns were named based on the food items with the highest factor loadings. We then calculated a standardized score for each participant by summing the consumption frequency for each food group and multiplying it by the factor loadings for each dietary pattern [25].

#### 2.5.2. Socio-Demographic Predictors of Dietary Patterns

We used multivariable linear regression to assess the associations between socio-demographic measures in 2013 and dietary intake pattern scores in 2015. We estimated standardized coefficients (ßeta) and 95% confidence intervals (CI). We identified potential confounders using directed acyclic graphs (DAGs) and by including in the model covariates of interest that had at least a 10% effect on the predictors of dietary patterns [26]. Variables of interest included education, income, occupation, and area of residence. The covariates modelled included age and an interaction term for education and income (education x income) to assess the potential modifying effect of income on the association between education and dietary pattern scores.

#### 2.5.3. Sensitivity Analysis

For each of the four dietary patterns, we found that a few individuals had very high consumption scores. To determine the potential impact that these participants might have on the effect estimates, we reassessed the association between the socio-demographic predictors and the four dietary patterns without these individuals 

#### 2.5.4. Sample Size

Sample size for this study was determined by considerations that led us to recruit ~1000 participants. Generally, for PCA, between five to ten participants per item will provide an adequate sample size (in our study 150–300 participants) [27]. As well, power calculations indicate that a sample of 500 participants (i.e., men or women) with at least 20% in each socio-demographic group allow us to detect a statistically significant and substantial difference between two mean dietary scores of at least 0.4 standard deviations with a two-sided 5% significance level, and 80% power.

### 2.6. Ethics Approval

Ethical approval for the study was obtained from Sukhothai Thammathirat Open University Research and Development Institute (protocol 0522/10 (approved in 2004)) and the Australian National University Human Research Ethics Committee (protocols 2004/344 (approved in 2004)), 2009/570 (approved in 2009) and 2015/068 (approved in 2015). Informed written consent was obtained from all participants. All data were de-identified before analysis. We thank Professor Aekplakorn for permission to use the Thai National Health Examination Survey FFQ.

## 3. Results

### 3.1. Participants

Of the 2400 randomly selected TCS participants, 1090 (45%) completed and returned their dietary surveys. Of these, 15 (10 men, 5 women) did not respond to >10% of their FFQ questions, so they were excluded. Analyses were based on the remaining 1075 participants and comparisons are summarised in the supplementary Table S2. Those who completed the FFQ and those who did not respond were similar with respect to body mass index (BMI), area of residence, occupation, and income (all *p*-values > 0.2), although respondents were, on average, older (*p* < 0.001) and had higher levels of attained education (*p* < 0.01).

When compared to the female participants, male participants were older, had a higher BMI, and earned a higher monthly income (*p* < 0.001). Male participants were also more likely to work as senior managers than female participants (*p* < 0.001). Having attained a university education was more common in women than in men (*p* < 0.01). These results were statistically significant but the actual differences were not large.

**Figure 1:**
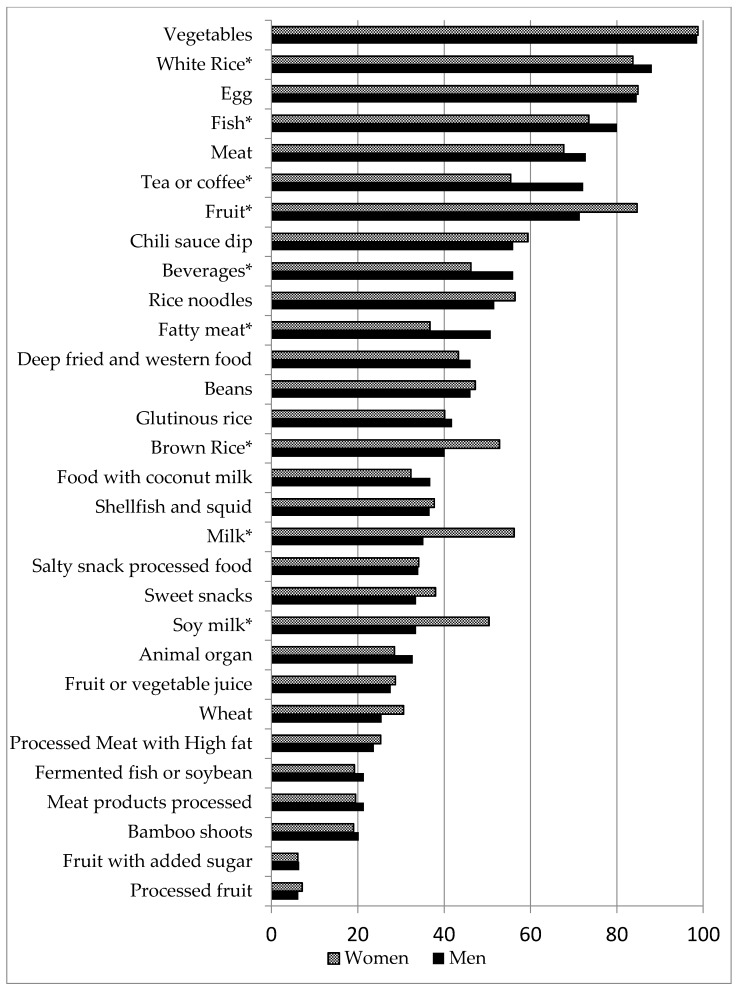
Percentage of Thai adults consuming each food group per week by sex. * χ^2^ *p* value < 0.05 when comparing weekly food group consumption frequency by sex.

Figure 1 shows the proportion of 2015 dietary survey participants consuming each food group per week by sex. Vegetables and white rice were the most commonly consumed food groups by all of the participants. As compared to women, men consumed higher proportions of white rice, fish, coffee, sugar-sweetened beverages, and fatty meat (*p* < 0.05). When compared to men, women consumed higher proportions of fruit, brown rice, milk, and soy milk (*p* < 0.05). 

### 3.2. Diet Patterns

Four dietary patterns were identified both in men and women (Table 1). These patterns are named here as Healthy Transitional (soy milk, beans (legumes), and milk (in both sexes), fruit (only in men) and fish (only in women)); Fatty Western (fatty meat and deep fried and western food (in both sexes), and meat (only in men)); Highly Processed (fruit with added sugar, processed fruit (in both sexes), sweet snacks and processed meat products (only in men), and salty snacks, wheat and juice (only in women)); and, Traditional (fermented fish and soybean, glutinous rice, bamboo shoots, and chili dipping sauce). The Healthy Transitional and Fatty Western diets are both characterized by high protein availability and increased dietary diversity while the Highly Processed and Traditional diets are both characterized by high sugar and starch availability and lower dietary diversity. The total variance explained by these four patterns in men and women, was 37% and 36%, respectively.

### 3.3. Socio-Economic Position and Dietary Patterns

The multivariable-adjusted sex-specific associations between SEP and the four dietary patterns are shown in Table 2 and Table 3. Among women, higher incomes of 20,001–30,000 or ≥30,001 Baht/month (vs. ≤10,000) were both associated with a lower Traditional diet score (ß = −0.62, 95% CI −1.06, −0.18) and (ß = −0.67, 95% CI −1.15, −0.19) and working as a manager or as a professional (vs. office assistant) were both associated with a lower Fatty Western diet score (ß = −0.60, 95% CI −1.14, −0.05) and (ß = −0.48, 95% CI −0.86, −0.11), respectively. Among men, a higher income was positively associated with a higher Healthy Transitional diet score, and working as a manual labourer was associated with a higher Fatty Western diet score; both ß coefficients were substantial (>0.5) but not statistically significant. 

In women, having a university education (vs. not) was associated with a lower Highly Processed diet score (ß = −0.57, 95% CI −0.98, −0.17) and a higher Traditional diet score (ß = 0.42, 95% CI 0.03, 0.81). In men, education level was not significantly directly associated with any of the dietary patterns. However, income modified the association between education and the Highly Processed diet (*p* for interaction 0.03). At a low income of <10,000 Baht per month, having a university education (vs. not) was associated with a lower Highly Processed diet score (ß = −1.02, 95% CI −1.78, −0.25).

### 3.4. Urbanization and Dietary Patterns

The associations between urban residence and the four identified dietary patterns are shown in Table 2 and Table 3. Among women, when compared to rural residence, urban residence was associated with a higher Fatty Western diet score (rural-urban: ß = 0.55, 95% CI 0.02, 1.08; urban-urban: ß = 0.68 95% CI 0.32, 1.04) and a higher Highly Processed diet score (ß = 0.44, 95% CI 0.13, 0.75,) but with a lower Traditional diet score (rural-urban: ß = −0.60, 95% CI −1.04, −0.17; urban-urban: ß = −0.68, 95% CI −0.98, −0.39). Among men, as compared to rural residence, urban residence was associated with a higher Fatty Western diet score (ß = 0.59, 95% CI 0.20, 1.00), and a lower Traditional diet score (urban-rural: ß = −0.77, 95% CI −1.39, −0.15; rural-urban: ß = −0.74, 95% CI −1.21, −0.26; urban-urban: ß = −1.00, 95% CI −1.31, −0.68).

### 3.5. Sensitivity Analysis

The effect estimates were similar when we removed the 15 individuals (nine men and six women) with the high consumption scores (see Methods) so these individuals were retained in the main analyses. Similarly, excluding individuals with missing data for any FFQ items did not change the results. 

## 4. Discussion

We assessed diets and their socio-demographic predictors in a prospective cohort of Thai adults. Using Principle Component Analysis, four major dietary patterns were evident: Healthy Transitional, Fatty Western, Highly Processed, and Traditional. For both sexes, high SEP associated with a lower consumption of unhealthy foods; urban residence associated with greater food diversity, but also with foods that have been shown to increase NCD risk in previous studies. 

Some limitations should be considered when interpreting our findings. The FFQ used in our study documented intake frequency and we did not adjust for energy intake. Another issue to consider is the subjective nature of decisions that is required by the factor analysis technique; although, this method is data driven, and at several points during the analysis the investigators are required to make important decisions [24]. These include the consolidation of individual foods into food groups, determining the number of factors to retain, choosing the rotation method, and labelling the factors in interpretable ways [24]. To minimize subjectivity, we used our knowledge of Thai cuisine and previous dietary pattern studies to guide our construction of food groups. Scree plots and eigenvalues supported the statistical basis for retention of four dietary patterns.

Important strengths of this study include the prospective data collection and nationwide coverage of our sample of Thai adults. We used an FFQ that has been previously validated in the national Thai population and which has been used to determine dietary patterns and their association with health outcomes [17]. Furthermore, the TCS participants are ideal for studying the association between socio-demographic factors and patterns of dietary consumption in LMICs since this cohort is becoming urbanized, using education for self-improvement, and experiencing the health-risk transition ahead of their fellow Thais [15].

The four dietary patterns identified in our study are similar to those reported in both LMICs and HICs, and the total variance explained by these factors in men and women (37% and 36%, respectively) is similar to what has been reported in previous studies [17,28]. The Fatty Western and Highly Processed dietary patterns in our study resemble the “Western”, “Unhealthy”, “Convenience”, or “Meat” diet patterns reported in LMICs [29,30] and HICs [13,31] since they are high in added sugars and saturated fat. These dietary patterns characterize the “degenerative” stage of the nutrition transition since they associate with increased NCD risk [3]. Some aspects of the Healthy Transitional pattern in our study resembles the “Prudent” or “Healthy” dietary patterns commonly reported in upper-middle income [11,32] or HICs [33]. This pattern reflects a shift from a traditional diet (carbohydrate based) to one that is high in dietary quality and diversity. Unlike the “Western Diet”, the Healthy Transitional pattern is associated with reduced NCD risk [33]. The Traditional dietary pattern is similar to the “Traditional” or “Carbohydrate” diet patterns reported in LMICs, with intakes high in dietary starches and low in dietary diversity [17,34].

In agreement with previous studies from upper-middle income countries [6,11,35] and HICs [13,36], we found that having a high SEP was associated with healthier and more diverse dietary patterns that reduce NCD risk. For example, women who earned a higher income were less likely to consume a traditional diet. Although the Traditional diet does offer various vitamins and minerals, it is also low in dietary diversity and high in starchy glutinous rice, which has been found to increase metabolic disease risk in Asian populations [17,37]. Although the association was not statistically significant, men who earned a higher income were more likely to consume a Healthy Transitional diet, characterized by the consumption of milk and brown rice, which are both associated with reduced risk of T2DM [38,39]. These findings support the nutrition transition theory that states that negative health behaviours reverse in the final stages of the transition and that this occurs first in high-SEP individuals [40]. Indeed, Thailand now has one of the highest gross national incomes (GNIs) among upper-middle income countries [14]. Younger Thais may already be exhibiting a “cultural resistance” to consuming western fast-food diets, with a higher resistance being reported among those with a higher education level [41]. Our findings highlight the need for public health efforts to target the promotion of healthy eating in low-SEP Thai adults.

Education was associated with the lower consumption of unhealthy dietary intake in both men and women, but in men, this effect was modified by income. Previous studies have found that income and education may have independent roles in dietary intake and health outcomes like obesity, and that in women, education has a stronger protective role than income [42,43,44]. In this cohort all of the participants had at least begun a university degree. Therefore, it may be that an independent association with education in men would only be apparent if there was larger variance in education levels between groups.

We found that female participants in this study consumed a lower proportion of high fat and highly processed foods (e.g., sugar sweetened beverages and fatty meat) than men. This finding has been consistently reported in the literature [11,45,46] and may be due to women’s concerns with weight loss and body size [47]. However, women tend to adopt health promoting behaviours and better health outcomes more rapidly than men [48]. This sex-specific finding also reflects what commonly occurs in middle-income countries as they progress along the nutrition transition. Such a difference in men and women is well-recognized and healthy eating should be promoted in Thai men.

In both sexes, urban residence was associated with consumption of a greater diversity of foods (e.g., higher meat consumption and lower rice consumption), but also with foods that have been shown to increase NCD risk in previous studies, a common finding in LMICs [3]. We also found that in Thai women, migrating from a rural residence to an urban residence was associated with consuming an unhealthy diet. This could relate to the greater availability of highly processed and unhealthy foods in urban areas that may not be as widely available in rural areas. Indeed, in Thailand, the association between urbanization and NCD-promoting dietary patterns has been attributed to growth of the modern food retail sector (e.g., western supermarkets, convenience stores) in urban areas [49]. Over the past two decades, the rapid growth of the modern food retail sector has led to a substantial decrease in the number of fresh markets that are available in urban areas, including Bangkok [49,50]. Unlike fresh markets that sell fresh foods, modern food retailers sell inexpensive and highly processed food items and these are considered to be “more fashionable” than the traditional Thai food retail sector [51]. Increasing access to affordable and healthy food in urban areas should be considered a priority as part of the national NCD control efforts.

## 5. Conclusions

In this prospective nationwide study of Thai adults, we found strong and coherent evidence that socio-demographic factors are associated with dietary patterns. Our findings suggest that Thai adults are exhibiting an increasingly “developed” country pattern of diets with an increasing SEP. Thai policy makers need to promote consumption of a healthy diet, particularly in urban residents, in men, and in low-SEP Thai adults as a central part of the national NCD control efforts, especially for the prevention of T2DM and cardiovascular diseases. 

## Figures and Tables

**Table 1:** Factor loadings * for four dietary patterns identified among Thai adults.

**Food Groups (Men)**	**Healthy Transitional**	**Fatty Western**	**Highly Processed**	**Traditional**
Soy milk	0.41	-	-	-
Beans	0.37	-	-	-
Fruit	0.34	-	-	-
Milk	0.32	-	-	-
Brown rice	0.30	-	-	-
Wheat	0.30	-	-	-
Fatty meat	-	0.38	-	-
Deep fried and western food	-	0.36	-	-
Meat	-	0.34	-	-
Rice noodles	-	0.33	-	-
Food with coconut milk	-	0.30	-	-
Fruit with added sugar	-	-	0.49	-
Processed fruit	-	-	0.44	-
Sweet snacks	-	-	0.38	-
Meat products (processed)	-	-	0.35	-
Fermented fish or soybean	-	-	-	0.53
Glutinous rice	-	-	-	0.47
Bamboo shoots	-	-	-	0.40
Chilli dipping sauce	-	-	-	0.33
Dietary variance explained %	10.9	10.8	8.5	6.7
**Food groups (Women)**	**Fatty Western**	**Healthy transitional**	**Highly processed**	**Traditional**
Deep fried and western food	0.35	-	-	-
Fatty meat	0.35	-	-	-
Food with coconut milk	0.31	-	-	-
Soy milk	-	0.37	-	-
Beans	-	0.37	-	-
Fish	-	0.36	-	-
Milk	-	0.30	-	-
Processed fruit	-	-	0.44	-
Wheat	-	-	0.34	-
Fruit or vegetable juice	-	-	0.33	-
Salty snacks	-	-	0.31	-
Fermented fish or soybean	-	-	-	0.49
Glutinous rice	-	-	-	0.47
Bamboo shoots	-	-	-	0.46
Chilli dipping sauce	-	-	-	0.31
Dietary variance explained %	11.2	9.7	7.8	7.1

* Only factor loadings >0.30 or <−0.30 are displayed in the body of the table. These represent correlation coefficients between individual food groups and each dietary pattern.

**Table 2:** Multivariable linear regression of socio-demographic predictors in 2013 and dietary intake pattern scores in 2015 in 486 Thai men.

Predictors	Beta Coefficients and 95% Confidence Intervals			
	Healthy Transitional	Fatty Western	Highly Processed	Traditional
**Income (Baht/month)**				
≤10,000	reference	reference	**	reference
10,001–20,000	−0.20 (−0.79, 0.40)	−0.09 (−0.68, 0.51)		0.06 (−0.39, 0.53)
20,001–30,000	−0.05 (−0.67, 0.57)	0.06 (−0.55, 0.68)		0.01 (−0.47, 0.49)
≥30,001	0.66 (−0.04, 1.36)	−0.16 (−0.86, 0.53)		−0.36 (−0.90, 0.18)
**Education**				
University	−0.35 (−0.82, 0.11)	−0.24 (−0.70, 0.22)		0.05 (−0.30, 0.41)
**Education level by income (Baht/month)**				
Below university	-	-	reference	-
<10,000, university	-	-	−1.02 (−1.78, −0.25)	-
10,001–20,000, university	-	-	0.07 (−0.54, 0.69)	-
20,001–30,000, university	-	-	−0.11 (−0.86, 0.64)	-
≥30,001, university	-	-	0.95 (−0.20, 2.09)	-
**Occupation**				
Manual worker	0.09 (−0.48, 0.67)	0.52 (−0.05, 1.09)	0.34 (−0.14, 0.82)	0.04 (−0.41, 0.48)
Office assistant	reference	reference	reference	reference
Skilled worker	0.26 (−0.44, 0.96)	0.19 (−0.50, 0.88)	0.26 (−0.32, 0.84)	−0.01 (−0.54, 0.54)
Professional	0.01 (−0.50, 0.51)	−0.07 (−0.57, 0.43)	0.03 (−0.39, 0.45)	−0.06 (−0.45, 0.33)
Manager	0.38 (−0.15, 0.92)	0.19 (−0.34, 0.72)	0.18 (−0.26, 0.63)	0.25 (−0.16, 0.66)
**Urban residence**				
Rural-rural	reference	reference	reference	reference
Urban-rural	−0.17 (−0.98, 0.63)	−0.18 (−0.98, 0.62)	−0.20 (−0.87, 0.46)	−0.77 (−1.39, −0.15)
Rural-Urban	0.44 (−0.18, 1.05)	0.29 (−0.32, 0.90)	0.24 (−0.26, 0.75)	−0.74 (−1.21, −0.26)
Urban-Urban	0.19 (−0.21, 0.60)	0.59 (0.20, 1.00)	0.14 (−0.19, 0.48)	−1.00 (−1.31, −0.68)

All Beta coefficients are adjusted for age and for each other. ** The *p* for interaction for education x income was statistically significant for the highly processed diet pattern and therefore the main effect associations between income and education with this pattern are not displayed.

**Table 3:** Multivariable linear regression of socio-demographic predictors in 2013 and dietary intake pattern scores in 2015 in 589 Thai women.

Predictors	Beta Coefficients and 95% Confidence Intervals			
	Healthy Transitional	Fatty Western	Highly Processed	Traditional
**Income (Baht/month)**				
≤10,000	reference	reference	reference	reference
10,001–20,000	−0.20 (−0.64, 0.24)	−0.01 (−0.45, 0.43)	−0.06 (−0.44, 0.31)	−0.20 (−0.55, 0.16)
20,001–30,000	−0.21 (−0.75, 0.33)	−0.22 (−0.76, 0.32)	0.26 (−0.20, 0.72)	−0.62 (−1.06, −0.18)
≥30,001	−0.37 (−0.96, 0.22)	0.03 (−0.56, 0.62)	0.48 (−0.01, 0.98)	−0.67 (−1.15, −0.19)
**Education**				
University	−0.02 (−0.51, 0.46)	−0.04 (−0.52, 0.44)	−0.57 (−0.98, −0.17)	0.42 (0.03, 0.81)
**Occupation**				
Manual worker	−0.22 (−0.76, 0.33)	−0.09 (−0.63, 0.46)	−0.15 (−0.61, 0.31)	−0.02 (−0.46, 0.42)
Office assistant	reference	reference	reference	reference
Skilled worker	0.18 (−0.69, 1.05)	0.09 (−0.78, 0.95)	0.05 (−0.68, 0.78)	−0.01 (−0.71, 0.69)
Professional	0.08 (−0.30, 0.47)	−0.48 (−0.86, −0.11)	0.06 (−0.26, 0.38)	0.08 (−0.23, 0.38)
Manager	0.28 (−0.26, 0.83)	−0.60 (−1.14, −0.05)	−0.13 (−0.59, 0.33)	0.26 (−0.18, 0.70)
**Urban residence**				
Rural-rural	reference	reference	reference	reference
Urban-rural	0.12 (−0.47, 0.70)	0.58 (−0.01, 1.16)	0.12 (−0.37, 0.62)	−0.22 (−0.69, 0.25)
Rural-Urban	0.08 (−0.46, 0.61)	0.55 (0.02, 1.08)	0.27 (−0.18, 0.72)	−0.60 (−1.04, −0.17)
Urban-Urban	−0.10 (−0.46, 0.27)	0.68 (0.32, 1.04)	0.44 (0.13, 0.75)	−0.68 (−0.98, −0.39)

All Beta coefficients are adjusted for age and for each other.

## Supplementary Materials

The following are available online at www.mdpi.com/2072-6643/9/11/1173/s1, Table S1: Food items and food groups derived from the food frequency questionnaire, Table S2: Participants versus non-participants in the 2015 TCS dietary survey.
