# Supplementary material for: Social Demography of Transitional Dietary Patterns in Thailand: Prospective Evidence from the Thai Cohort Study
Source: Nutrients. 2017 Oct 27;9(11):1173. doi: 10.3390/nu9111173 (PMC5707645; doi:10.3390/nu9111173)
Supplement: Supplementary file 1 [file nutrients-09-01173-s001.zip › nutrients-231885-supplementary.pdf]

Table S1: Food items and food groups derived from the food frequency questionnaire

Food group	FFQ food items
Meat	Beef, pork, chicken, duck meat without skin
Fatty meat	Beef, pork, chicken or duck with added skin
Processed meat (fatty)	Processed meat high in fat (i.e. sausage)
Processed Meat (salted)	Processed meat high in salt (i.e. dried/salted fish or meat)
Fish	Fresh and salt water fish
Shellfish and squid	Shrimp, shellfish, crab, squid
Animal organs	Liver from pig, duck or chicken
Egg	Egg
Beans	Legumes and their products (i.e. mung, soy, tofu)
White rice	White rice
Brown rice	Brown rice
Glutinous rice	Sticky rice
Rice noodles	Rice noodles
Wheat	White bread, wholemeal bread
Deep fried and western food	Deep fried pork, Pizza, hamburgers
Food with coconut milk	Curry style dishes (i.e. Tom Kha, red curry)
Fermented fish or soybean	Fermented salted fish, fermented beans
Chili sauce dip	Chilli sauces (i.e. nam prik)
Fruit	Sweet (i.e. Papaya, lychee) and non-sweet (i.e. green apple)
Fruit with added sugar	Candied fruit, fruit paste
Processed fruit	Dried, canned, pickled
Milk	Fresh milk, yoghurt,
Soy milk	Soy milk
Beverages	Sweetened milk, soft drink, energy drinks, sports drinks
Fruit or vegetable juice	Fruit or vegetable juice
Tea or coffee	Tea or coffee
Sweet snacks	Dessert (with sweet syrup, coconut milk, yolk), baked goods
Salt snacks (highly processed)	Potato chips, Instant noodles
Bamboo shoots	Bamboo shoots
Vegetables	Vegetables

Table S2: Participants *versus* non-participants in the 2015 TCS dietary survey

Characteristics in 2013	Non-participants*<br>n (%)	Participants*<br>n (%)	P value**
Total	1310 (55%)	1090 (45%)	
**Sex**			0.37
Men	572 (43.7)	496 (45.5)	
Women	738 (56.3)	594 (54.5)	
**Age years**			<0.001
Under 30	91 (7.0)	52 (4.8)	
30-39	629 (48.0)	450 (41.3)	
40 or over	590 (45.0)	588 (**54.0**)	
**Body Mass Index**			0.66
<23.0kg/m^2	632 (48.8)	548 (50.6)	
23.0-<25.0 kg/m^2	298 (23.0)	236 (21.8)	
$\geq$25.0 kg/m^2	366 (28.8)	300 (27.7)	
**Residence**			0.95
Rural	576 (44.4)	481 (44.5)	
Urban	721 (55.6)	599 (55.5)	
**Education**			<0.01
No university education	311 (23.9)	199 (18.3)	
University education	989 (76.1)	886 (**81.7**)	
**Occupation**			0.51
Manual worker	192 (16.2)	142 (14.6)	
Office assistant	421 (35.5)	323 (33.2)	
Skill worker	66 (5.6)	57 (5.8)	
Professional	328 (27.6)	294 (30.2)	
Manager	190 (15.2)	158 (16.2)	
**Income**			0.18
$\leq$10,000	291 (22.6)	246 (22.7)	
10,001-20,000	486 (37.7)	380 (35.1)	
20,001-30,000	305 (23.6)	246 (22.7)	
$\geq$30,001	208 (16.1)	210 (19.4)	

*Numbers may not add to total sample size due to missing responses for some characteristics

**χ^2 comparing each baseline characteristic by participation status
